# The Use of In-Situ Simulation to Improve Safety in the Plastic Surgery Office: A Feasibility Study

**Published:** 2014-01-09

**Authors:** Fred E. Shapiro, John B. Pawlowski, Noah M. Rosenberg, Xiaoxia Liu, David M. Feinstein, Richard D. Urman

**Affiliations:** ^a^Harvard Medical School, Beth Israel Deaconess Medical Center, Boston, Mass; ^b^University of Massachusetts Memorial Medical Center, Worcester; ^c^Harvard Medical School, Brigham and Women's Hospital, Boston, Mass

**Keywords:** in-situ simulation, simulation training, office-based surgery, plastic surgery, patient safety

## Abstract

**Objective:** Simulation-based interventions and education can potentially contribute to safer and more effective systems of care. We utilized in-situ simulation to highlight safety issues, regulatory requirements, and assess perceptions of safety processes by the plastic surgery office staff. **Methods:** A high-fidelity human patient simulator was brought to an office-based plastic surgery setting to enact a half-day full-scale, multidisciplinary medical emergency. Facilitated group debriefings were conducted after each scenario with special consideration of the principles of team training, communication, crisis management, and adherence to evidence-based protocols and regulatory standards. Abbreviated AHRQ Medical Office Safety Culture Survey was completed by the participants before and after the session. **Results:** The in-situ simulations had a high degree of acceptance and face validity according to the participants. Areas highlighted by the simulation sessions included rapid communication, delegation of tasks, location of emergency materials, scope of practice, and logistics of transport. The participant survey indicated greater awareness of patient safety issues following participation in simulation and debriefing exercises in 3 areas (*P* < 0.05): the need to change processes if there is a recognized patient safety issue (100% vs 75%), openness to ideas about improving office processes (100% vs 88%), and the need to discuss ways to prevent errors from recurring (88% vs 62%). **Conclusions:** Issues of safety and regulatory compliance can be assessed in an office-based setting through the short-term (half-day) use of in-situ simulation with facilitated debriefing and the review of audiovisual recordings by trained facilities inspectors.

Office-based surgery is one of the fastest growing segments of healthcare. In 2005, the American Society of Anesthesiologists estimated that at least 10 million office-based procedures were performed annually, twice the number of office-based surgeries performed in 1995.^1^ Historically, the majority of the office-based procedures involved cosmetic and reconstructive plastic surgery, ophthalmology, and gastroenterology. However, in the past 15 years the scope has expanded to multiple specialties. Patient morbidity and mortality outcomes associated with office-based procedures have been reported. While some studies have shown the incidence of both complications and death to exceed the rates at hospital-based practices, others have not. For example, Vila et al^2^ showed an increased complication and mortality rate that was later refuted by Coldiron et al^3^ and Morello et al,^4^ among others. Other studies to date have examined the impact of accreditation, provider qualifications, types of procedures and anesthesia/sedation on mortality and morbidity in the office.^5^^-^9 The 3 main accrediting agencies have presently accredited over 11,000 office-based surgical practices in the United States. These agencies are the Accreditation Association for Ambulatory Health Care (AAAHC), The Joint Commission (TJC), and the American Association for Accreditation of Ambulatory Surgery Facilities (AAAASF), with AAAHC being the most popular choice for over half of the accredited facilities.^10^^-^12 Relatively few offices are accredited by any of the nationally recognized agencies, mostly due to the fact that only 22 states require accreditation of office-based surgical practices, and only approximately 30 states even have some degree of regulation of office-based practices.^13^^,^^14^ Another factor is that many office-based facilities, when given a choice, simply choose not to pursue regulatory certification because of cost, labor, and time commitment.^15^^-^17 Although general policies in the healthcare industry have promoted patient safety-centered strategies, there is limited legislative mandate to employ these practices in the office-based setting, despite recommendations from several professional societies.^18^^,^^19^ Even those facilities that seek certification only need to demonstrate the presence of essential equipment and medications and the level of training of the staff.^12^

We hypothesized that to better gauge the ability of an office-based plastic surgery center to handle a medical emergency, we should simulate several medical emergencies in-situ and permit the clinic personnel to manage the events. We further hypothesized that simulation scenarios can contribute to increased awareness of safety issues by the office personnel. The advantages of in-situ simulation have been well described in hospital practices, ambulatory clinics, and recently, in office settings.^20^^,^^21^ The major advantages are the creation of realistic medical emergencies in the actual space and with the actual equipment and personnel of the office. In-situ scenarios are useful to conduct team training, perform evaluations of local processes, and uncover areas of inadequate patient safety responses.^22^

Much of the in-situ simulation has been utilized to advance the procedural responses of code teams and resuscitation personnel.^23^ Psychomotor skills have been shown to improve with repeated training sessions.^24^ Times to trigger responses are reduced and the ratings of various procedural skills improve with in-situ training at regular intervals.^25^ While individual benchmarks can be assessed, the overall scenarios do not focus on the specific issues that make an emergency at an office-based setting so problematic. The lack of medical personnel, the need to use lay personnel, the challenges that an event manager has to organize and manage, the logistical problems of being removed from laboratory, radiologic, and surgical facilities–these all present unique stresses in a time of crisis.

In this feasibility study, we present a series of in-situ simulations that were structured to assess these unique challenges, customized to meet the needs of the participants, and reviewed to ensure that the scenarios and debriefings met and amplified guidelines of common agencies of accreditation. One of the outcome measures is the results of the *AHRQ Medical Office Survey on Patient Safety* administered to all participants before and after exposure to two simulation exercises.^26^

## MATERIALS AND METHODS

### Design

With approval as an exempted study from the Beth Israel Deaconess Medical Center investigational review board, we conducted a needs assessment, planned pre- and postsurveys, designed scenarios, and structured debriefings to meet the needs of the office-based personnel and to conform to existing regulations of several accrediting agencies.

### Office setting and personnel

The in-situ simulations were conducted in 2 free-standing office-based surgical centers in the Boston area, both of which specialized in plastic surgery procedures. Each facility performed about 1000 procedures per year. These procedures were performed using both general anesthesia and deep sedation as well as conscious sedation techniques. Both centers had previously received nationally recognized accreditation and had incorporated various safety equipment, protocols, and standards as outlined by these accrediting agencies. All medications and equipment used in the scenarios, for both routine and emergency situations, were the property of the office and remained in their normal locations for all of the scenarios. The only exception to the usual routine was the presence of a human patient simulator (Laerdal SimMan, Stavanger, Norway) with its monitor/emulator in place of the usual vital signs monitor. In one case, the automated external defibrillator was altered to allow the conductive pads to clip onto the mannequin during defibrillation.

Office-based personnel consisted of reception staff, billing staff, managers, nurses, medical assistants, and surgeons. The level of training in emergency procedures was commensurate with each individual's professional training, although the lay office staff was current in their certification of basic life support (BLS).

### Needs assessment

A specific, office-based Needs Assessment was performed during 2 initial visits to the facility prior to the in-situ simulation session.^27^ To maintain the overall confidentiality of the staff as well as the secrecy of the nature of the simulations, only 1 surgeon and 1 nurse were involved in the initial visit. The general outline of the Needs Assessment form as well as specific requests from the office personnel helped to shape the eventual scope, topics, and debriefing focus for the simulation exercises, as shown in Table 1. A second visit was performed to convey a general schedule of events and to allow the simulation and audiovisual experts to confirm the location of the electrical outlets, camera positions, debriefing rooms, and projection equipment.

### Simulation design

Several scenarios were developed to address perceived knowledge deficits and to strengthen team dynamics during complex medical emergencies. These scenarios were based on both the general requirements determined by the Needs Assessment as well as the specific suggestions during the previsits. The scenarios were crafted to represent technically challenging tasks to the medical personnel as well as require urgent actions from the administrative staff members.^28^ In one scenario, for example, the patient experienced malignant hyperthermia (MH) during administration of a general anesthetic, as shown in Table 2. In another scenario, a surgeon and a surgical nurse were asked to excise a lipoma model and close a wound using subcuticular sutures. The lipoma model was conceived and built by our simulation experts and validated by local surgeons. In both scenarios, an actor represents the patient when checking in to the receptionist and while in the preoperative room.

Once in the procedure room, the patient was represented by the human patient simulator. The scenario in the procedure room was designed to consist of several stages, but the transition to each stage as well as alterations in the scenario could be managed by the simulation experts and were reflective of the actions performed by the office personnel. In general, each scenario lasted 20 minutes. The scenarios ended when sufficient therapeutic maneuvers had been performed or when the outside emergency medical teams had arrived to assume care for the patient.

Each scenario was followed by a structured debriefing. The debriefings were attended by all of the office personnel and had several overall goals (Table 3). All of the participants were asked to contribute to the discussion, which dealt with key concepts of Crisis Resource Management (CRM), video review of the scenario, issues of communication, and the role of each participant in maintaining the culture of safety in the office. At the end of the training session, all participants gave both verbal and written comments about the strengths and weaknesses of the simulator sessions.

### Evaluation

While individual performances were not assessed and no ratings were given, each debriefing did focus on examples of individual, team, and systemic problems that could impede rapid resolution of a crisis. Using video review of the scenarios, the debriefer could isolate specific instances of communication to either demonstrate useful examples or to encourage the group to suggest improvements in the choice of words or phrases. For example, team actions could be compared with existing ACLS (Advanced Cardiovascular Life Support) algorithms. The performance of certain tasks such as mask ventilation or chest compressions could be assessed for proper rate and chest excursion. Systemic issues such as the appropriate emergency telephone numbers, adequate written protocols, and explicit ingress and egress routes could be discussed in the context of the recorded scenario. Finally, a comparison between the performance of the office team could be made with existing benchmarks and specific recommendations of accrediting agency regulations.

Prior to the exposure to the 2 simulation exercises, all participants were administered the abbreviated version of the *AHRQ Medical Office Survey on Patient Safety*.^26^ This nationally validated survey tool is specifically designed to measure the culture of patient safety in medical offices from the perspectives of providers and staff. This survey is intended to measure to which extent the organization's culture emphasizes patient safety, teamwork, discussion of mistakes, and continuous improvement and learning. The same survey was then readministered after the completion of the 2 simulation exercises following the debriefing session.

### Statistical analysis of survey results

Responses from the survey were presented as frequencies and percentages. For the matched pairs, McNemar tests or Stuart-Maxwell tests were performed to examine the differences in participant survey responses before and after simulation sessions. The proportional differences among all respondents were compared using Fisher exact tests. All statistical tests were 2-sided, with a type I error of 0.05. A *P* value of less than 0.05 was considered to be statistically significant. All statistical analyses were performed with SAS version 9.3 (SAS Institute, North Carolina).

## RESULTS

### Overview

A total of 16 personnel in 2 plastic surgery offices participated in the half-day, in-situ simulation training exercise. After a brief introduction to the proposed schedule and a tour into the procedure room to meet a “healthy” mannequin on the operating table, the participants completed a safety culture survey and decided whether to allow for video recording of the sessions. The standardized (confederate) patient for the first scenario then arrived at the reception area and proceeded with the check in. The patient changed clothes and walked to the preprocedure area. One of the office nurses completed a screening questionnaire and physical examination. The surgeon and anesthesiologist then made their individual introductions, explanations and completed their own paperwork with the patient. The standardized (actor) patient then walked into the procedure room and a similarly dressed mannequin assumed the role of patient. After appropriate monitors were applied, the patient was prepped and draped for the procedure. A time out was performed. The specific stages of the scenario were then performed.

### Survey outcomes

Survey results were obtained from all participants who were administered the abbreviated version of the *AHRQ Medical Office Survey on Patient Safety*, both prior to case scenarios and after the completion of the 2 scenarios (Table 2) and the debriefing session (Table 3). The pre- and postintervention responses showed a statistically significant difference in a few categories, as shown in Table 4. Specifically, the participant survey indicated greater awareness of patient safety issues following participation in simulation and debriefing exercises in 3 areas (*P* < 0.05): the need to change processes if there is a recognized patient safety issue (100% vs 75%), openness to ideas about improving office processes (100% vs 88%), and the need to discuss ways to prevent errors from recurring (88% vs 62%). However, the rest of the survey responses did not show statistically significant differences between pre- and postintervention.

### Clinical outcomes

Two scenarios were demonstrated during the simulation exercise. The first was a toxic reaction to a local anesthetic injection, which resulted in ventricular tachycardia and pulseless electrical activity. The second case was a reaction to general anesthetics known as MH.^29^ In both cases, the anesthesiologist and surgeon needed to establish good communication and to orchestrate a larger response to the crisis. Clinical outcomes were deliberately varied and related to the speed of the response to ventricular tachycardia and the ability to assess the efficacy of therapy and the potential side effects from therapeutic intervention. For example, repeated doses of amiodarone would result in hypotension, whereas early cardioversion would lead to pulseless electrical activity. Thus, the participants were forced to reassess and try alternative therapies to better manage the patient. In the case of the MH patient, the vital signs were allowed to deteriorate until a full dose of 5 mg/kg of dantrolene was given. The timely delivery of such a large dose required the coordinated effort of between 3 to 8 people. Any delay in assembling the office staff to help with resuscitation resulted in longer periods of hypotension.

### Team-based processes

Team-based processes were evaluated according to the central principles of CRM as described by Gaba et al.^30^^,^^31^ Principles of Role Clarity, Communication, Personnel Support, Resources, and Global Assessment were outlined and described before the video debriefing.^32^ During the debriefing, the participants were encouraged to categorize the clinical elements and actions in terms of these principles of CRM and to suggest alternatives or improvements in words or actions. In all of the scenarios, there were issues relating to the recognition of a crisis, organization of a team leader, setting of priorities, and the provision of timely feedback. In some cases, the use of an algorithm (eg, ACLS) was used to compare actions against an accepted standard. In other cases, the algorithms were faithfully followed but one or several participants became overwhelmed by the number of clinical tasks and were not assisted by other, less busy members of the team. Individual skills and behaviors were reviewed to ensure that all participants stayed within their own level of training and scope of practice.^33^^,^^34^

### Systems-based issues

As team behaviors became bogged down, there appeared systems issues that contributed to the frustration and ineffectiveness of the participants. In the MH case, for example, there was a lack of large syringes and large bore needles in the accompanying kit. This lack of an adequate system slowed the rate at which dantrolene was able to be solubilized and administered to the patient. Also in the MH case, the lack of routine temperature monitoring frustrated the anesthesia provider and this lack of monitored temperature reading delayed the recognition of MH. While an increase in temperature is usually a late sign of MH, the participant ignored several early signs such as tachycardia, hypercarbia, and acidosis. Several minutes elapsed before the emergency was declared, and the appropriate emergency medical systems were activated.

Once activated, the medical systems worked with varying degrees of effectiveness. A prepared kit for the treatment of MH was already in the procedure room and there were enough vials of dantrolene, but not enough syringes to reconstitute and administer the medication. Nurses were unfamiliar with the medication, the diluent, and the initial and total doses. As a result, the anesthesia provider had to guide the nurses but, in turn, failed to recognize electrocardiographic signs of hyperkalemia and of ventricular ectopy. Several of the office staff left to procure more dantrolene, but the operating room personnel were unaware of their whereabouts or their progress. In general, the scenario suffered for lack of an event manager who was able to fully coordinate multiple activities.

During the debriefing, the office staff was able to identify further flaws in the systemic response to the emergency. After dialing 9-1-1, for example, the ambulance would arrive to the parking lot without knowing how to proceed to the procedure room. Even an enhanced ambulance emergency response system, such as the one that was available, does not provide directions within the building. The staff suggested that one of the reception staff would need to be in the parking lot to receive the ambulance and to direct the medical personnel. A route that would accommodate the width of a wheeled stretcher with a patient was devised. The need for periodic updates from the event manager in the procedure room to all the staff was emphasized, and the importance of the nonmedical personnel was reinforced.

### Generating recommendations

Following the scenarios and after a debriefing session that stressed the importance of leadership, communication, situational awareness, and elements of resuscitation, a number of specific recommendations were made to improve the process of crisis management (Table 5). These 5 recommendations came from open-ended questions in the follow-up survey given to the participants 1 month after the exercise. Three of the comments were centered on emergency algorithms that were discussed in the postscenario debriefings. Two of the comments related to continuation of deliberate practice of simulation and communication exercises. The speculated root-cause of these comments may have been derived from the group's performance in the simulation exercises and subsequent reflection on ways to improve.

All of the participants recognized the requirements for a coordinated manager of the medical emergency and for ready access to the written algorithms or protocols to help to plan for such an event. Most felt that their knowledge of ACLS was “rusty” and welcomed a refresher course or a series of simulations to periodically practice these resuscitative skills. While doctors and nurses were current with respect to ACLS certifications, the nonmedical office staff were not all current with BLS and felt that regular practice of BLS would be essential to be comfortable during a medical crisis.

In addition to the regular practice of appropriate ACLS and BLS algorithms, the office-based group discussed several other improvements in their systems-based approach to a medical emergency (Table 4). The office staff requested a regular review of the items in the code cart with a brief description of how they work and under what situation one might use them. The staff further asked for repeated simulation exercises to demonstrate other anticipated office-based problems. Finally, the office voiced a need to practice effective and closed-loop communication on a regular and nonurgent basis, to make it a habitual part of the working environment.

## DISCUSSION

While others have shown the utility of in-situ simulation^35^^-^37 as well as the feasibility of providing simulation training in an office-based setting,^23^ this report describes the novel use of in-situ simulation to establish the presence of necessary emergency items and procedures as required by the accrediting agencies, as well as to establish the workability of these items and procedures during a simulated medical emergency in a plastic surgery office. The actual office-based team was used to provide care during the crisis and the actions were recorded and professionally debriefed. In the course of the session, many examples of individual, group, and systemic behaviors were used to suggest possible improvements in both training and performance. Likewise, the video review allowed for the display of several actions that had the potential to affect patient safety, and these episodes were illustrated and discussed.

The in-situ nature of the simulation exercise allowed the office group to rehearse a response to an emergency with their own personnel and with their own equipment. The advantage of simulation when compared to a group discussion, for example, was that the personnel could physically handle the equipment and, thus, could detect any latent failures. These failures would not necessarily become clear during a discussion. The lack of sufficient syringes to mix the dantrolene, for instance, was an obvious systemic problem that became evident when the nurses had to actually mix the medication. We argue that these educational gaps causing systemic failures could have potentially been avoided through the use of a surgical safety checklist, which has been shown to increase safety and decrease cost in a hospital-based setting.^38^^,^^39^ Because the office-based setting presents unique challenges to patient safety, the Institute for Safety in Office-Based Surgery has implemented a surgical safety checklist specifically for the office setting.^40^

While the chosen scenarios represented both common occurrences in an office-based practice (medication side effect) as well as a rare, but potentially lethal condition (MH), the customized nature of the Needs Assessment does allow for the tailoring of the scenarios to the specific needs of the facility. For example, an office might want to practice a simulation of a recent adverse outcome that happened in their clinic or in the news. By modeling the scenario on an actual event, the staff can experience immediate appreciation or “buy-in” and can also relate their individual, team, and systems responses during simulation to an actual event. There is even the possibility to focus the debriefing on issues of individual performance (knowledge, skills, and attitude), team behavior (leadership and communication), and systems processes (available resources and systems activation).

Through slow motion review of recorded sessions, the debriefing can demonstrate that an emergency response does not occur until someone detects and declares an emergency. Furthermore, the evolution of a clinical problem into a full-blown crisis can be dissected during the debriefing. Latent failures can be demonstrated and preventative measures can be suggested that may be enough to avert a crisis. This ability to suspend time and to focus on the details is a distinct advantage of patient simulation with video review. This property can be utilized to improve the participant's understanding of the process with the hope of augmenting future participant actions.

The advantage of a debriefing performed by people with knowledge of the requirements of the accrediting agencies is that all actions can be reviewed with an eye to the specific requirements. The debriefer can attest to the office performance that is up to specific standards. Agencies form regulations and inspectors document compliance with the regulations, but in-situ simulation can confirm actions that adhere to the standards. In the automotive industry, there are requirements that car manufactures provide brakes and there are inspectors who confirm that the brakes work, but only a road test can demonstrate that the driver can apply the brakes at the right time and circumstance. Office-based, in-situ simulation offers the ability to “road test” the required equipment and personnel in their own vehicle (office) in a realistic situation. The simulation exercise can document exemplary individual, team, and systems performances and can identify items for improvement. While this type of simulation remains voluntary at present, one could envision a time when regulatory agencies might ask for such simulation-based demonstrations of compliance with their patient safety policies.

## FUTURE DIRECTIONS AND CONCLUSIONS

Issues raised during our simulation sessions can be explored further through similar simulation-based studies. Future investigations could be designed to assess knowledge and skills gaps, communication and organizational deficiencies, as well as system issues. We propose a number of potential lines of investigation. For example, knowledge of BLS and learning of these principles can be assessed using pre- and postexercise surveys. Basic life support motor skills, such as chest compression effectiveness, can be measured directly through the mannequin simulator, which can record compression rate, depth, and continuity. Communication skills such as percent closed-loop/read-back can be measured. Team training/CRM issues can be studied using video review with trained raters. Simulation scenarios can be designed to address teamwork, specifically assessing interactions between nurses, physicians, and nonmedical personnel. The adherence to checklists and algorithms for each of these parameters can also be measured. Systems issues can be explored through structured debriefings and surveys.

A recent article by Arriaga et al demonstrated the usefulness of surgical-crisis checklists, similar to the use of crisis checklists in the airline industry.^41^ The study used crisis checklists in a number of surgical-crisis scenarios in a simulated operating room environment and found that the checklist use was associated with significant improvement in the management of operating room crises. Specifically, 6% of steps were missed when checklists were available, versus 23% when checklists were unavailable. Interestingly, 97% of study participants reported that they would want the checklist used if they were undergoing an operation.^41^ This study provides good evidence to suggest that the use of similar checklists in patient care would potentially improve patient outcomes in the setting of operating room crises. Due to the exponential increase in the number of procedures performed outside the operating room, the need for further study of checklists in the outpatient setting continues. This issue was recently raised by Marjot et al. in the NEJM correspondence section.^42^ As checklists have been shown to be effective in the operating room and in ICU settings, their use must now be applied to care settings outside of the operating room. With the proper elements and planning, the checklist is a useful tool in patient safety.

In conclusion, issues of safety and regulatory compliance can be assessed in an office-based plastic surgery setting through the short-term (half-day) use of in-situ simulation with facilitated debriefing and the review of audiovisual recordings by trained facilities inspectors.

## Figures and Tables

**Table 1:** Needs assessment of plastic surgery office staff (outline)

Office-Based Surgery project
Why interested?
Lead (liaison) from the group
Needs assessment
Based upon what criteria?
Literature review
Common issues to think about:
Airway
Syncope
Bleeding
Infection
Local anesthetic toxicity
Experience
Problematic cases
Office staff needs training on Malignant Hyperthermia management
Group leadership would like the group to do a simulation-based exercise
Environment survey
Floor plan
Emergency preparedness
Equipment (emergency and non-emergency)
Policies
Personnel
Training
Curriculum Development
Scenario Topics
Medical issues
Team training
Emergency training
Systems analysis/planning
Pre- and Post-intervention survey
What types of questions?
Utilize validated questionnaire
Consent forms needed for IRB and video recording
Obtain IRB approval
Follow-up / Ongoing medical education
Return visits
Web-based services/content
Logistics of the project
Time needed for session
Half or full day? – prefer minimal interference with regular work schedule
Weekday or weekend?
Audio-visual device placement
Cameras
Microphones
Central or one for each person?
Debrief area—Main waiting room
TV screen available and seats for all participants
Portable simulation mannequin and A/V equipment
How to transport and set up
Time needed for setup and breakdown
How does this affect schedule?
Costs of the project (Preliminary discussion)
Overall
Pilot (Proof of concept)
Non-Pilot
Personnel
Equipment
Curriculum prep time
Follow-up
ACLS training

**Table 2:** Sample scenario template: malignant hyperthermia

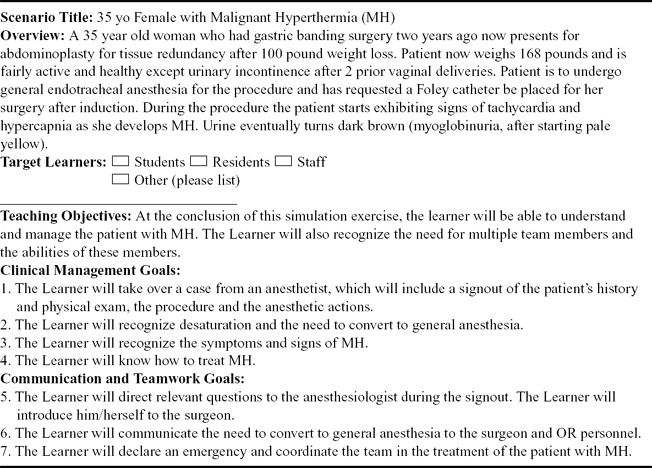

**Table 3:** Debrief outline

• Case Review
– Reactions Phase
• Feelings
• Facts
– Understanding
• Exploring
– Crisis Resource Management (CRM) perspective
– Clinical perspective
– Summary
• Reflecting
• Generalizing
• Applying
Crisis Resource Management Principles
• Role Clarity
• Communication
• Support
• Resources
• Global Assessment

**Table 4:** Survey responses pre- and postsimulation and debriefing exercises

Variable	Pre (N = 8)	Post (N = 8)	*P*
When there is a problem in our office, we see if we need to change the way we do things, n (%)			.03*
. Disagree or Strongly Disagree	0 (0%)	2 (25%)	
. Agree or Strongly Agree	8 (100%)	6 (75%)	
Mistakes happen more than they should in this office, n (%)			.16
. Disagree or Strongly Disagree	7 (88%)	5 (63%)	
. Neither Agree nor Disagree	1 (13%)	1 (13%)	
. Strongly Agree	0 (0%)	2 (25%)	
It is just by chance that we don't make more mistakes that affect our patients, n (%)			.11
. Disagree or Strongly Disagree	8 (100%)	6 (75%)	
. Agree or Strongly Agree	0 (0%)	2 (25%)	
This office is good at changing processes to make sure the same problems don't happen again, n (%)			.223
. Strongly Disagree or Disagree	1 (13%)	2 (25%)	
. Neither Agree nor Disagree	2 (25%)	0 (0%)	
. Agree or Strongly Agree	5 (63%)	6 (75%)	
The wrong chart/medical record was used for a patient, n (%)			.07
. Strongly Disagree or Disagree	7 (88%)	7 (88%)	
. Agree or Strongly Agree	1 (13%)	1 (13%)	
A patient's chart/medical record was not available when needed, n (%)			.75
. Strongly Disagree or Disagree	6 (75%)	4 (50%)	
. Agree or Strongly Agree	2 (25%)	4 (50%)	
Medical information was filed, scanned, or entered into the wrong patient's chart/medical record, n (%)			.45
. Strongly Disagree or Disagree	5 (63%)	6 (75%)	
. Agree or Strongly Agree	3 (38%)	2 (25%)	
Medical equipment was not working properly or was in need of repair or replacement, n (%)			.51
. Strongly Disagree or Disagree	5 (63%)	5 (63%)	
. Neither Agree nor Disagree	2 (25%)	1 (13%)	
. Agree or Strongly Agree	1 (13%)	2 (25%)	
A critical abnormal result from a lab or imaging test was not followed up within 1 business day, n (%)			.51
. Strongly Disagree or Disagree	5 (63%)	5 (63%)	
. Neither Agree nor Disagree	1 (13%)	2 (25%)	
. Agree or Strongly Agree	2 (25%)	1 (13%)	
When someone in this office gets really busy, others help out, n (%)			.37
. Strongly Disagree or Disagree	1 (13%)	0 (0%)	
. Neither Agree nor Disagree	1 (13%)	0 (0%)	
. Agree or Strongly Agree	6 (75%)	8 (100%)	
In this office, there is a good working relationship between staff and providers, n (%)			.37
. Strongly Disagree or Disagree	1 (13%)	0 (0%)	
. Neither Agree nor Disagree	1 (13%)	0 (0%)	
. Agree or Strongly Agree	6 (75%)	8 (100%)	
In this office, we often feel rushed when taking care of patients, n (%)			.51
. Strongly Disagree or Disagree	6 (75%)	5 (63%)	
. Agree or Strongly Agree	2 (25%)	3 (38%)	
In this office, we treat each other with respect, n (%)			.13
. Strongly Disagree or Disagree	1 (13%)	2 (25%)	
. Agree or Strongly Agree	7 (88%)	6 (75%)	
Staff in this office are asked to do tasks they haven't been trained to do, n (%)			.37
. Strongly Disagree or Disagree	7 (88%)	4 (50%)	
. Neither Agree nor Disagree	0 (0%)	1 (13%)	
. Agree or Strongly Agree	1 (13%)	3 (38%)	
Providers in this office are open to staff ideas about how to improve office processes, n (%)			.02*
. Strongly Disagree or Disagree	0 (0%)	1 (13%)	
. Agree or Strongly Agree	8 (100%)	7 (88%)	
Staff are afraid to ask questions when something does not seem right, n (%)			.22
. Strongly Disagree or Disagree	4 (50%)	6 (75%)	
. Neither Agree nor Disagree	3 (38%)	0 (0%)	
. Agree or Strongly Agree	1 (13%)	2 (25%)	
Providers and staff talk openly about office problems, n (%)			.37
. Strongly Disagree or Disagree	2 (25%)	5 (63%)	
. Neither Agree nor Disagree	1 (13%)	0 (0%)	
. Agree or Strongly Agree	5 (63%)	3 (38%)	
In this office, we discuss ways to prevent errors from happening again, n (%)			.02*
. Strongly Disagree or Disagree	1 (13%)	3 (38%)	
. Agree or Strongly Agree	7 (88%)	5 (62%)	

*Indicates statistically significant result (*P* < 0.05).

**Table 5:** Summary of suggested improvements by office staff following simulation and debriefing exercises

Review BLS/ACLS algorithms
Have algorithms in the OR
Have regular code cart review
Repeated simulation exercises
Practice team communication regularly
